# Tumor-derived exosomes regulate expression of immune function-related genes in human T cell subsets

**DOI:** 10.1038/srep20254

**Published:** 2016-02-04

**Authors:** Laurent Muller, Masato Mitsuhashi, Patricia Simms, William E. Gooding, Theresa L. Whiteside

**Affiliations:** 1 University of Pittsburgh Cancer Institute, Pittsburgh, PA 15213, USA; 2 Department of Otolaryngology and Head & Neck Surgery, University Hospital in Basel, CH 4031 Basel, Switzerland; 3 Hitachi Chemical Research Center, Inc., Irvine, CA 92617, USA; 4 FACS Core Facility, Loyola University School of Medicine, Maywood, Il 60153, USA; 5 Division of Biostatistics, University of Pittsburgh Cancer Institute, Pittsburgh, PA 15213, USA; 6 Departments of Pathology, Immunology and Otolaryngology, University of Pittsburgh School of Medicine, Pittsburgh, PA 15261, USA

## Abstract

Tumor cell-derived exosomes (TEX) suppress functions of immune cells. Here, changes in the gene profiles of primary human T lymphocytes exposed *in vitro* to exosomes were evaluated. CD4^+^ Tconv, CD8^+^ T or CD4^+^ CD39^+^ Treg were isolated from normal donors’ peripheral blood and co-incubated with TEX or exosomes isolated from supernatants of cultured dendritic cells (DEX). Expression levels of 24–27 immune response-related genes in these T cells were quantified by qRT-PCR. In activated T cells, TEX and DEX up-regulated mRNA expression levels of multiple genes. Multifactorial data analysis of ΔCt values identified T cell activation and the immune cell type, but not exosome source, as factors regulating gene expression by exosomes. Treg were more sensitive to TEX-mediated effects than other T cell subsets. In Treg, TEX-mediated down-regulation of genes regulating the adenosine pathway translated into high expression of CD39 and increased adenosine production. TEX also induced up-regulation of inhibitory genes in CD4^+^ Tconv, which translated into a loss of CD69 on their surface and a functional decline. Exosomes are not internalized by T cells, but signals they carry and deliver to cell surface receptors modulate gene expression and functions of human T lymphocytes.

Exosomes are virus-size (30–150 nm in diameter) membrane-bound vesicles secreted by normal as well as malignant cells and are present in all body fluids^1^,^2^. Tumor cells are avid producers of exosomes, and tumor-derived exosomes (TEX) have been reported to carry molecules and factors able to suppress functions of immune cells^3^,^4^,^5^. TEX have also been reported to induce activation and expansion of human regulatory T cells (Treg) and myeloid-derived suppressor cells (MDSC) *in vitro* and *in vivo*^6^,^7^,^8^. Given the emerging insights into the role of the host immune system in cancer progression, considerable attention is being directed at TEX and their direct and indirect effects on immune cells.

Patients with cancer, especially those with advanced disease, often have depressed anti-tumor immunity^9^,^10^. The mechanisms responsible for immune suppression in cancer and for tumor escape from the immune system have been extensively investigated and shown to be mediated by a variety of mechanisms, which may be tailored to individual tumors [reviewed in^11^]. TEX, which carry a spectrum of membrane-bound and soluble factors, many of which have been shown to mediate immune suppression, represent yet another mechanism utilized by tumors to subvert anti-tumor functions of immune cells^3^,^12^. Presumably, this mechanism is strictly dependent on the type of molecular cargo exosomes carry and the presence of relevant receptors on immune target cells. For example, we have previously reported that TEX inhibited functions of human activated CD8^+^ T lymphocytes by inducing their apoptosis via the Fas/FasL pathway^13^,^14^. Direct effects of TEX carrying a highly biologically-active membrane-form of FasL (42 kDa) upon co-incubation with CD95^+^ activated CD8^+^ T cells included down-regulation of signaling mediated by the T cell receptor (TcR), cytochrome C release from mitochondria, a loss of the mitochondrial membrane potential (MMP), caspase-3 cleavage and DNA fragmentation^3^,^14^,^15^. Interestingly, these effects of TEX could be in part blocked by pre-incubation of human T cells with IRX-2, a cocktail of natural cytokines^14^. In *in vitro* experiments and upon administration to patients with cancer as a therapeutic, IRX-2 was effective in protecting human CD8^+^ T cells from TEX-mediated apoptosis^14^. Protection of the immune cells from TEX-induced dysfunction and death, inhibition of suppressive signaling by TEX or both are likely to become important aspects of future therapeutic anti-tumor strategies^16^,^17^. For this reason, a better understanding of cellular and molecular mechanisms TEX utilize to mediate immune suppression is necessary.

Current approaches to overcoming tumor-induced suppression of anti-tumor T cell activity depend on the use of check-point inhibitors, such as, e.g., antibodies (Abs) specific for CTLA-4, PD-1 or PD-L1^18^,^19^. The ongoing clinical trials with these checkpoint inhibitors provide evidence that a therapeutic restoration of anti-tumor responses can be successful in improving outcome for some patients with cancer^20^. Consequently, there is much interest in identifying other molecular pathways contributing to tumor-induced immune suppression and potentially in silencing of these pathways. TEX carry a wide range of suppressive molecules derived from the tumor cell surface and the cytoplasm of the parental tumor cell^1^,^2^,^3^,^21^. So armed, exosomes can interact with immune and non-immune cells delivering signals which specify suppression of essential functions in the responder cells. TEX have been reported to be able to modify the transcriptional profile of the recipient cells such as human brain microvascular endothelial cells or human hematopoietic cells^22^,^23^. In view of these reports, we considered the possibility that TEX-delivered signals induce changes in the transcriptional profile of T cells and that the immune response-regulating genes would be preferentially targeted in T lymphocytes, especially in activated T lymphocytes. The objective of this study is to demonstrate that TEX co-incubated with freshly purified human CD4^+^ CD39^+^ Treg, conventional CD4^+^ T cells (CD4^+^ Tconv) or CD8^+^ T lymphocytes differentially regulate expression of the key immune function-related genes in these T cell subsets.

## Results

**Figure 1:**
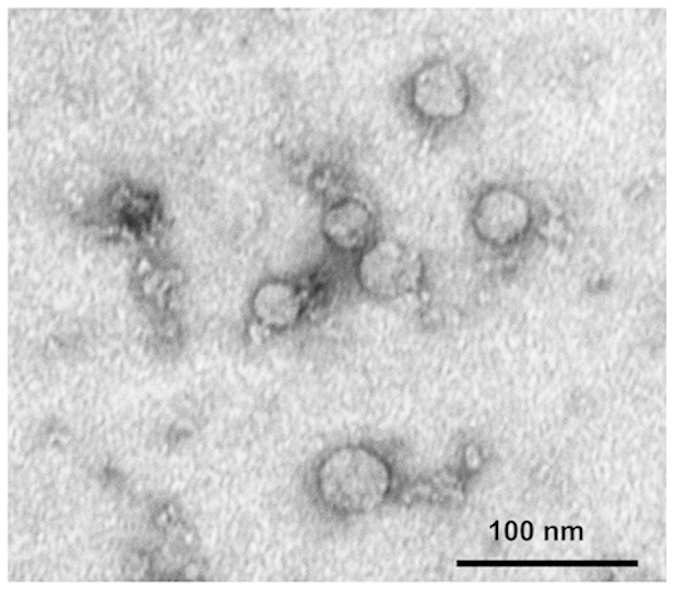
Transmission electron microscopy (TEM) of exosomes isolated from supernatants of PCI-13, a HNSCC cell line. Exosomes isolated by differential centrifugation, ultrafiltration and size exclusion chromatography were placed on copper grids, stained with uranyl acetate and examined. Note their vesicular morphology and the size range, which does not exceed 50 nm. The TEM image was acquired and generously provided by Dr. Sonja Funk.

Exosomes isolated from supernatants of the PCI-13, a human tumor cell line, or dendritic cells (DC) had the expected morphology by TEM (Fig. 1), the particle size in the range of 30–100 nm by NanoSight and were biologically active in NK-cell assays as previously described by us^24^. Immunobead-based capture of CD4^+^ Tconv, CD8^+^ T cells and CD4^+^ CD39^+^ Treg from normal donors’ PBMC by AutoMACS yielded highly enriched subsets of T cells to be targeted by exosomes. Isolated CD4^+^ and CD8^+^ T cell subsets had the purity of over 90%, while the purity of CD4^+^ CD39^+^ Treg varied from 80 to 85%, as determined by flow cytometry.

### Effects of TEX on mRNA profiles in resting vs. activated T cell subsets

CD4^+^ T cells (CD4^+^ Tconv), CD8^+^ T cells and CD4^+^ CD39^+^ Treg were isolated from peripheral blood of three normal donors and each isolated subset was individually co-incubated with exosomes isolated from supernatants of cultured tumor cells (TEX) or from supernatants of cultured human dendritic cells (DEX). In preliminary titration experiments, we observed that TEX-induced changes in lymphocyte mRNA expression were exosome dose dependent, cell type dependent and cell activation dependent. For example, Supplemental Figure 1 shows that the Ct values for *IL-8* mRNA expression levels were not changed by TEX in resting or activated CD4^+^ Tconv or CD8^+^ T cells, while in activated CD4^+^ CD39^+^ Treg, TEX significantly increased the Ct value for *IL-8* (i.e., they down-regulated the *IL-8* gene expression level), but only at one TEX to Treg ratio (1 ugTEX protein/25,000 Treg). These preliminary experiments indicated that TEX-induced changes in mRNA gene profiles need to be independently evaluated in *resting* and *activated* T cell subsets using calibrated TEX doses and pre-determined numbers of target cells to achieve optimal effects. Based on these and other preliminary experiments, we also concluded that a higher numbers of T cells would be needed to optimize the mRNA recovery. Therefore, in all subsequent experiments larger numbers of resting as well as activated (via the T cell receptor) T cells (0.5 × 10^6^ to 1 × 10^6^/well) were co-incubated with TEX at the constant exosome concentration of 10 ug protein or in PBS.

### Effects of TEX and DEX on mRNA expression levels in T cells

**Figure 2:**
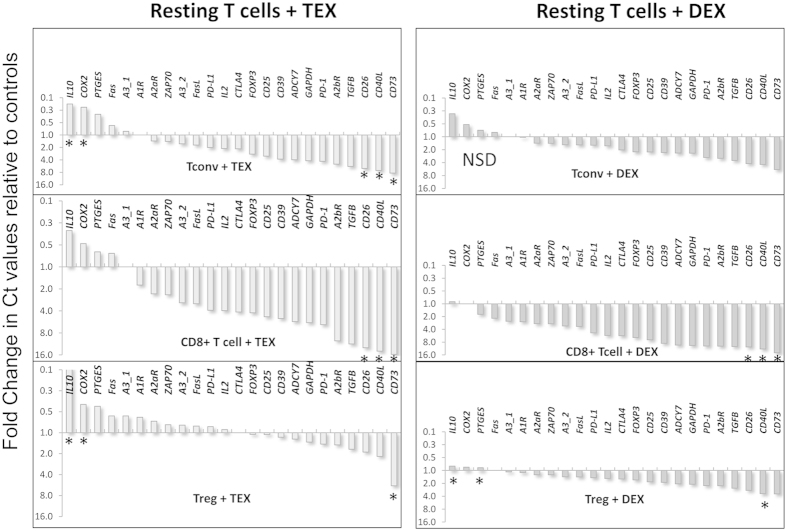
Waterfall plots for fold changes in mRNA expression levels for 24 genes in freshly-isolated (resting) CD4^+^ Tconv, CD8^+^ T cells and Treg. T cells incubated in parallel with PBS were used as controls. The data are for responding T cells of 1/3 normal donors. The data are calculated as fold changes in Ct values relative to Ct values of controls and are presented using an inverted scale based on the principle that high Ct values correspond to lower mRNA expression levels. Note quantitative differences in mRNA expression profile for T cells co-incubated with TEX vs. DEX. Asterisks indicate significant differences in gene expression (p = <0.05).

**Figure 3:**
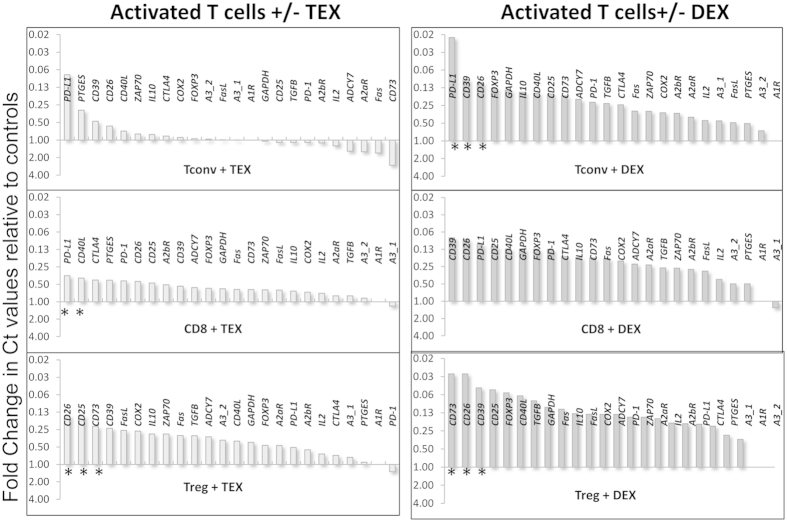
Waterfall plots for fold changes in mRNA expression levels for 24 genes in activated T cell subsets after co-incubation with TEX or DEX. The data for the responder T cells of the same donor (1/3) who donated resting T cells (see Fig. 1) are presented using an inverted scale, as described in the legend to Fig. 1. Note up-regulation of gene expression in activated T cells co-incubated with TEX and DEX. Asterisks indicate significant differences in gene expression (p = <0.05).

While all exosomes isolated from supernatants of tumor cell lines (TEX) are tumor derived, those isolated from supernatants of cultured human dendritic cells (DEX) are produced by normal cells. To evaluate effects of exosomes on mRNA gene expression in lymphocytes, we established a model *in vitro* system comprised of isolated allogeneic TEX or DEX co-incubated with human T cell subsets (CD4^+^ Tconv, CD8^+^ T and CD4^+^ CD39^+^ Treg) for 16 h. T cells were isolated from peripheral blood of three different randomly- selected normal donors. Following co-incubation with TEX or DEX, cellular mRNA was harvested from T cells, reverse transcribed and analyzed by qRT-PCR in the microplate system described in Materials and Methods. Changes in expression levels of the selected 24–27 genes in T cells were simultaneously measured relative to the levels in control wells (PBS; no TEX or no DEX). The waterfall plots in Figs 2 and 3 illustrate fold changes in expression levels of these genes in the three T cell subsets (resting or activated) of one representative normal donor.

In *resting* CD4^+^ Tconv, fold changes in mRNA expression levels were similar for TEX and DEX (Fig. 2). With the exception of only 4 genes (*IL-10, COX-2, PTGES* and *Fas*), expression levels of all other genes were decreased relative to controls. Only few of these decreases were significant, including expression of *CD26*, *CD40L* and *CD73*. Resting CD8^+^ T cells were more responsive to TEX or DEX than CD4^+^ Tconv. Treg were least responsive to TEX or DEX and showed a distinct change in the mRNA profile after co-incubation with TEX. Expression of *IL-10* and *COX-2* was significantly up-regulated, while that of *CD73* was significantly down-regulated by TEX. All other genes were not significantly altered in expression.

In *activated* T cells, the mRNA expression levels were up-regulated relative to controls by both TEX and DEX but these changes were quantitatively different, with DEX inducing greater transcriptional increases than TEX in nearly all genes in all three T cell subsets (Fig. 3). This was not a consistent result, however, as with the third donor’s T cells, down-regulation of gene expression levels was seen, similar to that observed in resting T cells. Activated Treg appeared to be more responsive to TEX than the other two T cell subsets. Interestingly, in activated Treg, the genes coding for CD25 (IL-2R), ectonucleotidases (CD39 and CD73) and adenosine deaminase (CD26) were significantly up-regulated in expression by either TEX or DEX. Also, the *PD-L1* expression levels were up-regulated in CD4^+^ Tconv and CD8^+^ T cell but less so in Treg. In aggregate, the data suggest that activated T cells are highly susceptible to transcriptional modulation by both TEX and DEX, and that the initial T cell activation level might determine up-or down-regulation in gene expression, with poorly-activated T cells behaving like their resting counterparts.

### Multifactorial data analysis

To determine significance of the observed fold changes in cellular mRNA expression levels, multifactorial analysis of the data for all T cell subsets obtained from three different donors and incubated with TEX or DEX was performed. By calculating and combining mean ΔCt values for all factors (T cell subsets, T cell activation, exosome source or exosome absence) and using normalized mean ΔCt for all tested genes, we determined that of the three factors considered, it was cellular activation followed by the responding T cell type that best discriminated between exosome-mediated effects on mRNA expression levels in lymphocytes (Table 1). The exosome source had little or no impact on fold changes of gene expression levels in T cells co-incubated with TEX or DEX.

### Heat-map analysis

**Figure 4:**
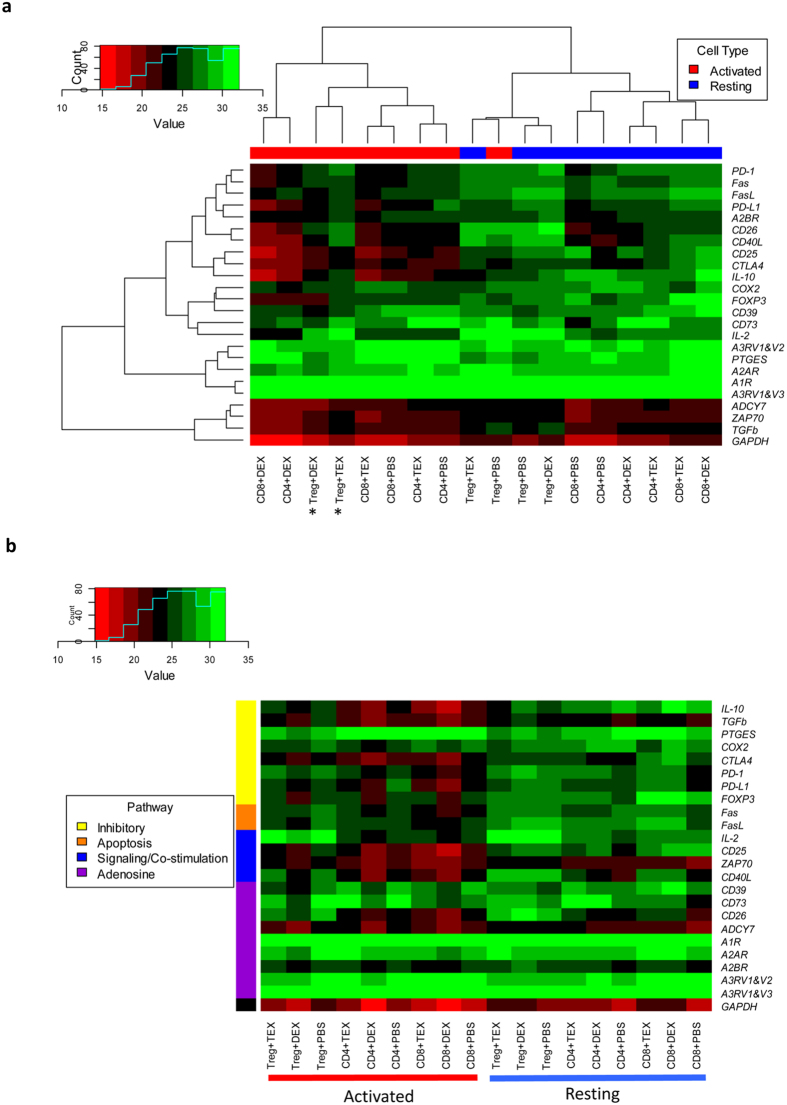
Unsupervised analysis (**a**) and supervised analysis (**b**) of the combined data obtained with different resting and activated T cell subsets of 3 normal donors co-incubated with TEX or DEX. The data are mean Ct values calculated for mRNA expression levels of each of the 24 genes examined. In (**a**) note higher Ct values (i.e., lower mRNA expression levels) for a number of immunoregulatory genes in activated T cell subsets. The asterisks indicate differences in gene expression of activated Treg co-incubated with TEX or DEX. In (**b**) the same data for resting and activated T cell subsets are compared in a supervised analysis to indicate mean Ct values for genes regulating inhibitory, stimulatory, apoptotic or adenosine-related pathways.

To be able to compare exosome-induced changes in expression levels of individual genes within the T cell subsets, unsupervised and supervised heat-map analyses were next performed. An unsupervised heat map for the entire data set (Fig. 4A) illustrates differential effects of TEX and DEX on activated vs. resting T cells. Two major clusters were identified. Within the *activated* T cell cluster, TEX and DEX induced distinct transcriptional changes in T cells, as indicated by lower Ct values (i.e., higher level of mRNA transcription) for DEX- vs TEX-induced transcripts, as also shown by waterfall plots in Fig. 3. The heat map indicates that gene expression changes induced by TEX or DEX are quantitatively different from those in PBS-treated control T cells. Activated Treg co-incubated with TEX have a distinct transcriptional profile from that seen in Treg incubated with DEX (see asterisks in Fig. 4A). The lowest transcriptional activity (in green) occurred for the adenosine pathway-related genes, and the highest (in red) in the immunoregulatory genes such as *PD-L1, PD-1, CD40L, CD25, ZAP-70*. Within the *resting* T cell cluster, all three T cell subsets co-incubated with TEX, DEX or PBS, show minimal or no changes in transcriptional activity as does *GAPDH*, which is equally highly expressed in controls and after co-incubation with exosomes.

To further compare changes in expression levels of individual genes induced by TEX vs DEX in different T cell subsets, a supervised heat map was constructed, in which the selected 24 genes were grouped according to the molecular pathways they regulate (Fig. 4B). Again, *resting* T cells were minimally affected by co-incubation with TEX or DEX. Among the *activated* T cell subsets, DEX induced higher transcriptional changes than TEX, especially in genes involved in the inhibitory (*IL-10, TGF-β, CTLA-4, PD-1, PD-L1*) and signaling (*Zap70, CD40L, CD25, CD26*) pathways. Expression levels of genes regulating adenosine receptors and ectonucleotidases, CD39 and CD73, were not up-regulated following co-incubation of T cells with DEX or TEX. Interestingly, activated Treg were less susceptible to transcriptional changes mediated by TEX than the other two T cell subsets. The supervised heat map suggests that exosomes exert differential effects on genes involved in molecular pathways operating in activated in T cells.

### Effects of TEX on human CD4^+^ CD39^+^ Treg

**Figure 5:**
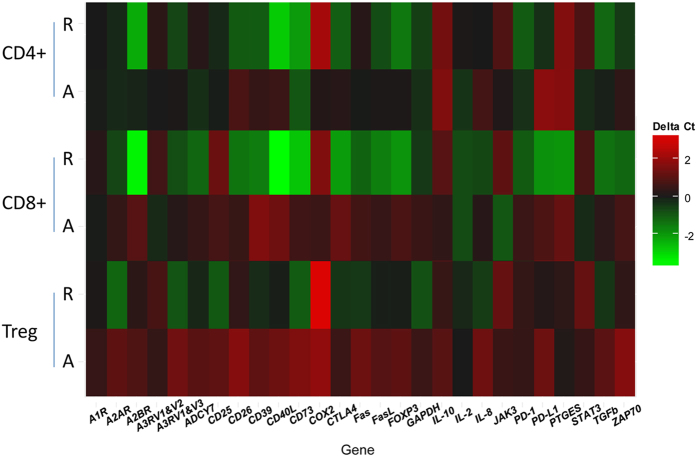
A supervised heat map for mRNA changes in resting (**R**) and activated (**a**) T cell subsets of 3 different normal donors after T cell co-incubation with TEX for 16 h at 37 °C. Control CT values were obtained for the same cells co-incubated with PBS alone. The data are mean ΔCt values for all 3 T cell donors. The higher ΔCt values (in red) in activated T cells (especially in activated Treg) reflect higher gene expression levels relative to PBS. Note that nearly all genes in activated Treg co-incubated with TEX gave higher ΔCt values than those obtained for the same genes in the other T cell subsets.

Given that Treg were previously shown by us to respond to TEX by *in vitro* expansion and increase in suppressor functions^6^, translational profiles of CD4^+^ CD39^+^ Treg were compared to those of CD4^+^ Tconv or CD8^+^ T cells after their co-incubation with TEX. A heat map was constructed which displays mean ΔCt values for 27 genes (*IL-8, JAK3* and *STAT3* were added) following co-incubation with TEX of resting or activated CD4^+^ Tconv, CD8^+^ T and Treg cells obtained from all three donors (Fig. 5). High ΔCt values (in red) denote increased mRNA levels relative to PBS controls after exposure to TEX, and as expected, *activated* T cells, especially activated Treg, show positive ΔCt values for nearly all tested genes. There were some notable gene changes in T cells co-incubated with TEX: the expression levels of *COX-2* and *IL-10* were increased in all subsets of resting and activated T cells, and more genes were up-regulated in CD4^+^ CD39^+^ Treg than in CD4^+^ Tconv. Results in this heat map, combining mRNA measurements for all three T cell donors, are consistent with the waterfall plots shown in Fig. 3 for one representative donor. Compared to other T cell subsets, CD4^+^ CD39^+^ Treg had broader and higher transcriptional activity after co-incubation with TEX. This suggests that activated Treg are more susceptible to TEX-mediated regulation of mRNA expression levels than CD8^+^ T cells or CD4^+^ T conv.

### Exosome interactions with immune cells

**Figure 6:**
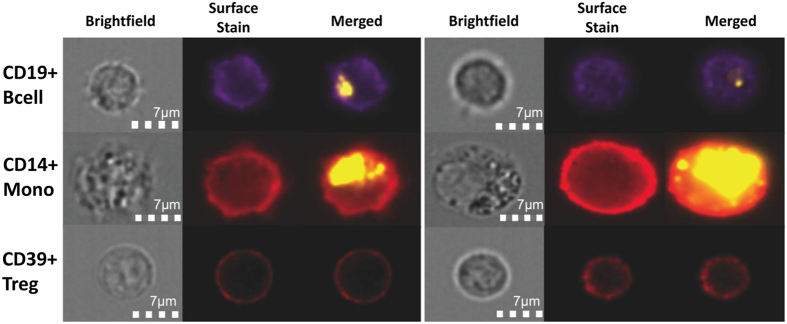
Uptake of PKH26-labeled TEX by human mononuclear cell subsets. TEX were labeled with the PKH26 dye as described in Methods and co-incubated for different time periods with freshly isolated human CD19^+^ B cells, CD14^+^ monocytes and CD39^+^ Treg. Shown are images of these cells (captured independently in two different fields) in a brightfield, after staining for the distinguishing surface markers and after uptake of labeled exosomes. Note that Treg did not internalize exosomes.

To further investigate cellular interactions responsible for exosome-induced changes in the gene expression profile of immune cells, we labeled TEX with PKH26 dye and monitored their uptake by T cells, B cells and monocytes isolated from human peripheral blood. Image analyses using an Amnis Image Stream cytometer showed that CD14^+^ monocytes and CD19^+^ B cells readily took up and internalized PKH26^+^ TEX during 24 h of co-incubation. Surprisingly, resting or activated Treg (or conventional CD4^+^ and CD8^+^ T cells; data not shown) did not internalize TEX even after 72 h of co-incubation (Fig. 6). These results indicated that in T cells, TEX internalization was not necessary for delivery of signals that result in changes of gene expression, and suggest that surface-mediated receptor-ligand interactions might be sufficient for inducing the observed changes.

### Functional analyses of T cells co-incubated with TEX

**Figure 7:**
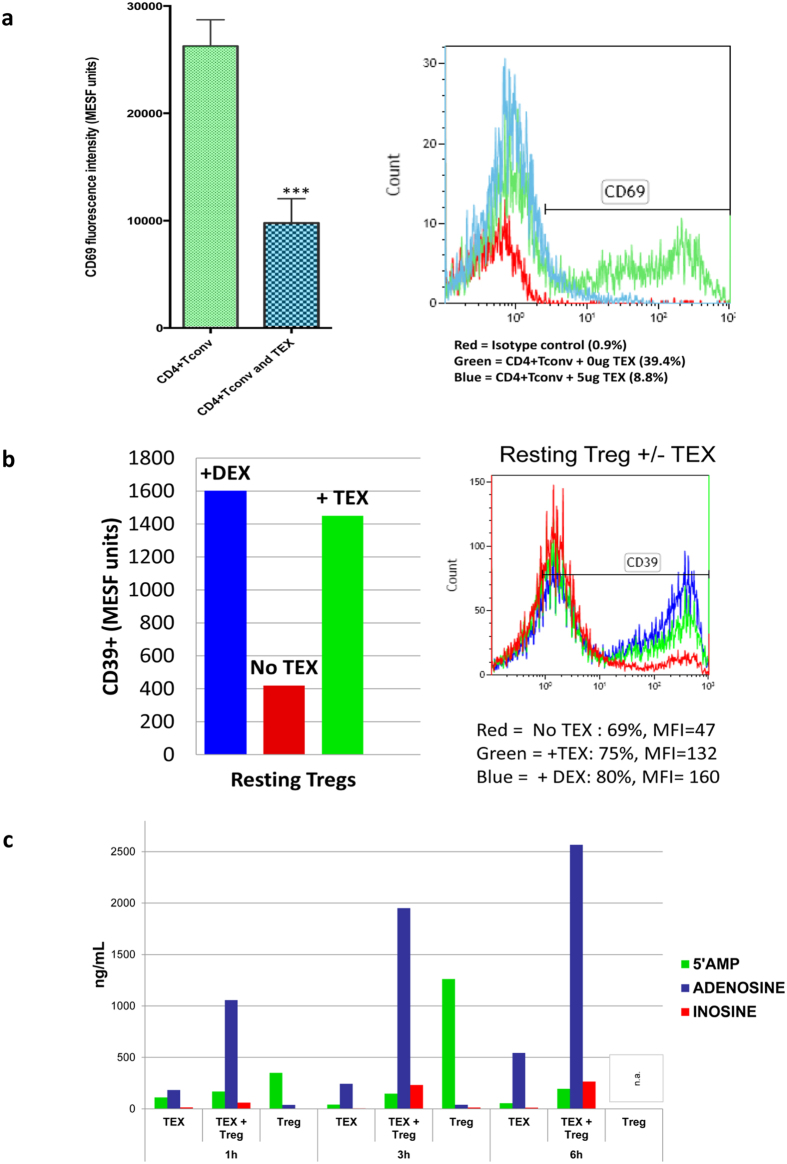
Effects of TEX on protein expression and functions of T cells. In (**a**) down-regulation of CD69 protein expression on the surface of responder CD4^+^ Tconv after co-incubation with TEX. Activated CD4^+^ Tconv were co-incubated with TEX (10 ug protein) produced by the PCI-13 cells or with PBS for 12 h. The CD69 expression levels on CD4^+^ Tconv were then determined by flow cytometry (MFI) and were converted into MESF units based on calibration curves established with fluorescent calibration beads. The bar graphs show data (mean values ± SD) from 3 independent experiments performed with CD4^+^ Tconv obtained from different normal donors. The asterisks indicate p values at p < 0.0005. In (**b**) changes in expression levels of CD39 protein on the surface of resting CD4^+^ CD39^+^ Treg co-incubated with TEX produced by the PCI-13 cell line or DEX. The exosomes were used at the concentration of 10 ng protein/ assay. Exogenous ATP was added as described in Methods. Flow cytometry (*right*) shows up-regulation of MFI for CD39 in a representative experiment, and the bar graph summarizes results of three experiments performed with Treg obtained from different donors. In (**c**), Production levels of 5′ AMP, adenosine and inosine by resting CD4^+^ CD39^+^ Treg co-incubated with TEX produced by the PCI-13 cell line. The data are from one of two experiments performed in the presence of exogenous ATP. The analyte levels were measured by mass spectrometry as described in Methods.

To demonstrate that TEX-induced changes in the transcriptional profile of activated T cells have functional consequences, we activated normal CD4^+^ Tconv with anti-CD3/CD28 Abs in the presence of IL-2 and after co-incubation with TEX, determined expression levels (MFI) of the CD69 protein (an activation marker) on the surface of these cells by flow cytometry. As shown in Fig. 7A, TEX significantly (p = 0.0005) down-regulated expression levels of the CD69 protein in activated CD4^+^ Tconv, suggesting that TEX interfered with T cell activation. Viewed in the context of the above-presented evidence for the elevated expression levels of genes encoding proteins involved in suppression such as COX_2_, CTLA-4, Fas, FasL or TGF-β in activated CD4^+^ Tconv co-incubated with TEX (see Fig. 5), we surmise that TEX selectively enhanced mRNA expression and its translation into inhibitory proteins which interfered with CD4^+^ T cell activation as evidenced by a decrease in CD69 protein levels. These data are consistent with our previous reports of TEX inducing immune suppression in activated T cells (13–15).

Focusing on effects exerted by TEX on Treg, we examined expression of proteins involved in the adenosine pathway, which is used by Treg to operate suppression (28, 29). The Anova analysis of our data indicated that exosomes induced significant change in expression levels of the genes involved in the adenosine pathway (Table 1). The levels of mRNA encoding CD39 and CD73, CD26 and adenylate cyclase-7 were down-regulated upon co-incubation of resting Treg with TEX or DEX (Fig. 2). To determine whether these exosome-induced changes in mRNA expression levels translated into protein changes in Treg, we next co-incubated the same TEX with freshly-isolated resting CD4^+^ CD39^+^ Treg in the presence of exogenous ATP. We examined: (a) changes in expression of CD39 on the Treg surface and (b) adenosine production by these Treg. As shown in Fig. 7A,B, co-incubation of Treg with TEX significantly increased expression levels of CD39 and adenosine production by these cells. It also increased expression levels of intracytoplasmic CD79 in these cells (data not shown). Our data suggest that TEX- mediated down-regulation of mRNA coding for adenosine pathway genes in Treg translates into a burst of enzymatic activity leading to immunosuppressive adenosine production and thus enhanced suppressor functions.

## Discussion

Our earlier studies of human T cells co-incubated with TEX or exosomes isolated from plasma of patients with cancer showed that these nanovesicles down-regulated CD3ζ and JAK3 expression in primary activated T cells and mediated Fas/FasL-driven apoptosis of activated CD8^+^ T cells^3^,^13^,^14^,^15^,^25^. TEX also promoted proliferation of CD4^+^ Tconv and their conversion into CD4^+^ CD25^high^ FOXP3^+^ CD39^+^ Treg^6^,^12^, which co-expressed IL-10 and TGF-β, CTLA-4, granzyme B/perforin and effectively mediated immune suppression^26^,^27^,^28^,^29^. In experiments reported by us and others, TEX were also shown to interfere with functions of NK cells and monocytes^3^,^30^,^31^,^32^,^33^. These *in vitro* studies of suppressive effects of TEX on functions of human immune cells are supported by *in vivo* studies in mouse models, where TEX were shown to suppress anti-tumor immune functions and promote tumor progression^34^,^35^. In aggregate, these data suggest that TEX represent a mechanism used by tumors to escape from the host immune system.

We suspected that TEX could serve as the vehicle responsible for inducing changes in mRNA expression levels in T cells. To study exosome-induced alterations in mRNA of responder T cells, we used a model system comprising isolated subsets of human primary T cells co-incubated with TEX which were exclusively derived from cultured tumor cells. Exosomes produced by cultured human dendritic cells (DEX) originated from normal, non-cancerous hematopoietic cells. This is an allogeneic model system, in which T cell responses could be biased in part by their alloreactivity with exosomes carrying MHC molecules. Also, it is an artificial system in which exosomes derived from cell lines rather than exosomes isolated from human body fluids are used. In our hands, the initial co-incubation experiments performed with plasma-derived exosomes from patients with cancer and normal donors (data not shown) gave inconsistent results, which appeared to be exosome-donor related, presumably because exosomes obtained from plasma are mixtures of vesicles originating from many different cells. Hence, we resorted to the *in vitro* model system for TEX and DEX, in which the source and characteristics of exosomes were well defined and uniform. By TEM and NanoSight, these extracellular vesicles fit with the definition adopted for exosomes^1^,^16^. A highly sensitive method was needed for reliable detection of changes in gene expression levels using mRNA extracted from a small number of primary T cells co-incubated with exosomes. This was especially important when working with CD4^+^ CD39^+^ Treg, which represent <5% of human circulating CD4^+^ T cells^27^, and were available in limited quantities. The subset of CD4^+^ CD25^hi^FOXP3^+^ Treg co-expressing CD39 is commonly present in the circulation of patients with cancer and is referred to as inducible (i) Treg^26^,^29^. We used a qRT-PCR method developed by Mitsuhashi *et al*. that was previously successfully applied to the analysis of human leukocyte functions^36^,^37^. As previously reported, an increase in the Ct values of less than two-fold was often statistically significant using this method, especially for abundantly expressed genes^36^,^37^. Using the model, we expected to gain evidence for a direct, target-cell specific transfer of molecular signals delivered by TEX that initially involves mRNA synthesis and/or translation and ultimately leads to functional dysfunction of immune cells such as occurs in cancer^9^,^10^.

Exosomes are known to deliver miRNA species to cells^23^,^38^, and TEX derived from cultured glioblastoma cells have been reported to be able to modify the mRNA expression profile of the recipient fibroblasts^22^. In view of these reports, we expected that changes in mRNA expression levels would be selective, that they would be distinct in CD4^+^ Tconv vs. Treg and that DEX, serving as surrogates for exosomes derived from non-malignant cells, would induce different mRNA profile changes in T cells than TEX. In particular, we expected to show that TEX primarily induced alterations in expression of genes regulating immune suppression. Instead, we found that TEX and DEX similarly modulated mRNA expression levels, inducing decreases in resting and increases in activated T cells. Changes in expression levels of immunoregulatory genes such as *COX*_*2*_*, IL-10, CD39, CD73, PDL-1 or CD26* were significant in T cells co-incubated with TEX or DEX. The transcriptional changes induced by exosomes were not restricted to any specific mRNA species but were evident in multiple genes regulating inhibitory, apoptotic, signaling/co-stimulatory or adenosine-associated pathways (Fig. 4B). In activated T cells, these changes were quantitatively somewhat smaller upon co-incubation with TEX than DEX. The multivariate analysis of the data generated with cells of the three different donors identified factors that significantly influenced mRNA gene expression levels in target cells exposed to exosomes as: (a) the presence/absence of exosomes; (b) the T cell activation level; and (c) the type of responding T cells. In contrast, the exosome source (TEX or DEX) was not a significant discriminating factor in the model.

In addition to cellular origins of exosomes, their interactions with the target cell may be critical for information transfer. Depending on the nature of the target cell, exosomes may be readily or not so readily internalized^39^. While phagocytic cells rapidly take up exosomes, and in cultured human brain microvascular endothelial cells, green fluorescent protein (GFP)-labeled exosomes can be seen in the cytosol within hours of co-culture^22^, our results with PKH26-labeled TEX showed that T cells, even activated T cells, do not internalize TEX (Fig. 6). Therefore, we concluded that in T cells, exosomes deliver signals to receptors present on the cell surface, which ultimately result in alterations of the mRNA profile. In contrast to B cells and monocytes, which internalized exosomes and enabled transfer of miRNAs, Treg co-incubated with TEX even for 72 h did not internalize exosomes. In the absence of the cytosolic protein/ nucleic acid transfer, cell surface signals delivered by TEX to T cells were translated into alterations in mRNA expression levels, which clearly had functional consequences, as shown by down-regulation of the CD69 protein expression on the surface of activated CD4^+^ Tconv cells or increased adenosine production by resting Treg co-incubated with TEX (Fig. 7C).

Different subsets of activated T cells seemed to respond differently to TEX, and the heat map in Fig. 4 shows that TEX induced quantitatively and qualitatively distinct effects in CD4^+^ Tconv than in CD4^+^ CD39^+^ Treg: the ΔCt values for nearly all 27 genes examined were higher in activated Treg than in other T cells, especially in activated CD4^+^ Tconv. This finding suggests that activated Treg (an equivalent of induced Treg or pTreg in humans) may be more sensitive to TEX-mediated effects than other T cells. Also, the gene profile of activated CD4^+^ Tconv co-incubated with TEX indicates low expression levels of genes regulating immune suppression, e.g., *COX2, CTLA-4, Fas, FasL, TGF-β* (Fig. 5). Our data do not indicate whether TEX modulate mRNA synthesis or mRNA translation into proteins. However, given that low expression levels of these and other genes in activated CD4^+^ Tconv co-incubated with TEX correlates with the significantly lower MIF of the CD69 protein on the surface of these cells, we suggest that TEX inhibit activation of CD4^+^ Tconv by promoting translation of the genes encoding inhibitory proteins.

TEX-mediated effects on Treg were distinct from those observed in CD4^+^ Tconv. In resting Treg, TEX induced higher CD39 expression and adenosine production, while downregulating mRNA expression levels of the genes regulating this immmunosuppressive pathway (Figs 2 and 7B,C). Considering that resting Treg need to be activated or induced to efficiently mediate suppression, TEX appear to be able to deliver such activating signals, leading to increased CD39 and CD73 expression and production of adenosine in resting Treg. In contrast, in activated Treg, where gene transcription and translation are likely to be efficient, co-incubation with TEX or DEX induced up-regulation in expression of the same immuno-suppressive genes. This observation suggests that TEX also exert distinct effects on resting vs. activated Treg. Given our previous functional data on TEX-mediated suppression in immune cells^3^,^13^,^14^,^15^, we speculate that TEX-induced up-regulation of the inhibitory gene expression levels in activated Treg promotes their rapid translation into inhibitory proteins. Co-incubation with TEX increases levels of critical immunoinhibitory proteins, such as TGF-β, IL-10, COX-2 as well as CD39, CD73 and adenosine production. Our *ex vivo* studies of iTreg in the peripheral circulation and tumor sites of patients with HNSCC illustrated significant overexpression of CD39 and CD73 ectoenzymes in these cells^12^,^26^. Because plasma of these patients contains elevated levels of exosomes, including TEX, relative to NCs plasma^12^,^24^, it is tempting to associate this iTreg phenotype with TEX-mediated effects. Overall, our studies provide evidence for differential exosome-mediated alterations in gene expression levels in resting vs activated T cells and support the role of TEX in differential modulation of gene expression and T cell functions in CD4^+^ Tconv vs Treg.

## Methods

### Peripheral blood mononuclear cells (PBMC)

Buffy coats obtained from normal volunteers were purchased from the Central Blood Bank of Pittsburgh. Mononuclear cells were recovered by centrifugation on Ficoll-Hypaque gradients (GE Healthcare Bioscience), washed in AIM-V medium (Invitrogen, Grand Island, NY, USA) and immediately used for experiments.

### Isolation of the peripheral blood T-lymphocyte subsets

T cell subsets were isolated via an immunoaffinity-based capture procedure, using Miltenyi beads as previously described^26^. Negative selection to isolate CD4^+^ T cells was followed by the separation of CD4^+^ CD39^+^ and CD4^+^ CD39^neg^ T cells using anti-CD39 Ab-coated Miltenyi beads by AutoMACS. The purity of the isolated cells was determined by flow cytometry. The isolated T cell subsets were either directly used for experiments (resting T cells) or activated by incubation in the presence of anti-CD3/anti-CD28 antibody (Ab)-coated beads and IL-2 (150 U/ml) for 4 h or overnight, depending on the experiment. To confirm activation, cells were harvested, stained for CD69, and the frequency of CD69^+^ T cells as well as CD69 expression (MFI) on the cell surface were determined by flow cytometry. The MFI values were converted into MESF units, based on fluorescent intensity curves generated with calibration beads.

### Isolation of exosomes

Exosomes were derived from: (a) supernatants of the head and neck squamous cell carcinoma (HNSCC) cell line, PCI-13 maintained in a long-term culture^40^. This cell line served as a source of TEX; (b) supernatants of human dendritic cells (DC) cultured from monocytes isolated from PBMC by adherence to plastic and incubated in the presence of IL-4 and GM-CSF^41^ for 4 days. These supernatants were used as a source of DC-derived exosomes (DEX). The DC cultured from plastic-adherent monocytes were > 90% CD40^+^ CD83^+^ CD86^+^ DR^+^ by flow cytometry. Media used for cell cultures contained FCS which was ultracentrifuged at 100,000 for 3 h to deplete it of bovine exosomes. Exosomes isolated from other HNSCC cell lines (PCI-1, PCI-30) as well as exosomes isolated from plasma of patients with HNSCC and of normal donors as previously described^24^ were also used in preliminary experiments to establish their effects on mRNA transcription and on inhibition of T cell functions (data not shown). Supernatants were routinely concentrated in a Vivacell prior to exosome isolation^42^. Exosomes were isolated as described by us previously^24^. Briefly, differential centrifugation (1.000 xg for 10 min at 4 °C and 10,000 xg for 30 min at 4 °C) was followed by ultrafiltration (0.22 μm filter; Millipore, Billicera, MA, USA) and then size-exclusion chromatography on a A50 cm column (Bio-Rad Laboratories, Hercules, Ca, USA) packed with Sepharose 2B (Sigma-Aldrich, St. Louis, MO, USA). The exclusion volume fractions were collected, ultracentrifugated (100,000 xg for 2 hr at 4 °C), and pellets were resuspended in phosphate buffered saline (PBS). Protein concentrations of exosome fractions were determined using a BCA Protein Assay kit as recommended by the manufacturer (Pierce, Thermo Scientific, Rockford, lL-61105, USA).

### Characterization of isolated exosomes

Prior to co-incubation with T cells, isolated exosomes were evaluated for morphology by transmission electron microscopy (TEM), particle distribution and size in a NanoSight instrument and biological activity by flow cytometry to demonstrate their ability to down-regulate NKG2D expression in isolated human NK cells as previously described^24^. TEM of isolated exosomes was performed at the Center for Biologic Imaging at the University of Pittsburgh as previously described (24). Briefly, freshly-isolated exosomes were put on a copper grid coated with 0.125% Formvar in chloroform. The grids were stained with 1% (v/v) uranyl acetate in ddH_2_O, and the exosome samples were examined immediately. A JEM 1011 transmission electron microscope was used for imaging.

### Flow cytometry

The phenotype of freshly-isolated primary T cell subsets was evaluated by multiparameter flow cytometry. Cells were incubated in the dark for 20 min at room temperature (RT) with a panel of labeled monoclonal antibodies (mAbs): anti-CD4-eFluor 450 (48-0048-42, eBioscience, CA, USA), CD8-ECD (6604728, Beckman Coulter, Brea, CA, USA), CD39- PC7(25-0399-42, eBioscience) PD-1-PE (12-2799-42, BioLegend), CD25-APC/Cy7 (302613, BioLegend, San Diego, CA, USA); CD69 FITC (555530, BD Biosciences, San Jose, CA, USA) and Foxp3-APC (17-4776-41, eBioscience). Isotype controls were used for each experiment. After incubation, cells were again washed, resuspended in flow buffer and analyzed using Gallios flow cytometer (Beckman Coulter). At least 5 × 10^4^ events were collected, and the data were analyzed using Kaluza software (Beckman Coulter). For fluorescence quantitation standardized fluorochrome microspheres were used as recommended by the company (Quantum^TM^ FITC-5 MESF, 555, Bangs Laboratories, Fishers, Indiana, USA).

### Labeling of exosomes with the PKH26 dye

Isolated exosomes (up to 200μg protein) were added to wells containing 2 mL of PKH26 (10^−6^ M in diluent buffer) and after 60 seconds, the staining reaction was stopped by adding 7 mL of PBS. Exosomes were pelleted by ultracentrifugation at 100,000 ×g for 2 h. They were then re-suspended in PBS at the concentration of ∼1 μg protein/μL and used for co-incubation with immune cells.

### Amnis-based imaging of immune cells

Lymphocyte subsets and monocytes were isolated from PBMC of NCs using AutoMACs. T lymphocytes were incubated in a medium containing 150 IU/mL of IL-2 (Miltenyi Biotech Inc,) and 150 uL of microbeads coated with anti-CD3/CD28 Abs (Miltenyi). B cells were activated using CD40L and IL-4 as previously described^12^. Monocytes were activated with 10 ng/mL of LPS (Sigma Aldrich, St. Louis). All cells were activated for 24 h. They were then aliquoted at 10^6 ^cells/mL/well into wells of 24-well plates and co-incubated with PKH26-labeled exosomes for various time periods. Cells were harvested, washed and stained for surface markers with PE-labeled antibodies specific for CD4, CD8, CD39, CD19 or CD14 as described above. Image analysis was performed using an Amnis cytometer. Cells were first visualized in a brightfield, identified as T cells (CD4^+^, CD8^+^, CD39^+^), B cells (CD19^+^) or monocytes (CD14^+^) based on surface staining and observed for the presence of intracytoplasmic PKH26. The cell images were merged to confirm uptake of PKH26 by individual cells.

### Co-incubation of resting or activated T cells with exosomes prior to mRNA isolation

Isolated T cells (resting or activated for 4 h) were seeded in wells of a 96-well plate at the cell concentration of 1 × 10^6 ^per mL in triplicate. Isolated exosomes (50–100 μg protein in initial titration experiments) were added to each well and the plate was incubated for 16 h at 37 °C. Control wells contained no cells or no exosomes. Following extensive washing in PBS to remove exosomes, T cells were used for mRNA isolation.

### mRNA isolation and qRT-PCR analysis

T-lymphocyte subsets co-incubated with TEX, DEX or PBS were dispensed into individual wells of a 96-well filter plate (0.5–1.0 × 10^6 ^cells per well). Cells were lysed and the lysates were transferred to an oligo (dT)-immobilized microplate for capture of poly (A)^+^ RNA. Next, cDNA was synthesized using target gene-specific antisense primers cocktail on the plate as previously described^36^,^37^. In this system, cDNA primed by specific primers comes to solution, while oligo (dT)-primed cDNA remains immobilized on the plate. Reverse transcription PCR of the solubilized specifically primed cDNA follows. In the absence of reverse transcriptase, no amplification takes place in this system^36^,^37^. Following reverse transcription, samples were analyzed by qRT-PCR assays in 96-well plates for expression of 24–27 immune response-related genes listed in Supplementary Table 1. The table also lists the primer sequences used. For PCR, the initial temperature cycle of 95 °C for 5 min was followed by 45 cycles of 95 °C for 30 sec, 65 °C for 1 min in a final volume of 5 μL of 1× SYBR green PCR master mix (Bio-Rad, Hercules, CA, USA) in a 384-well plate. The cycle threshold (Ct) was determined by the analytical software (SDS, Applied Biosystems). A Ct of 32 was used as a baseline. All samples were analyzed in triplicate and all samples for a single experiment were tested simultaneously. Results are expressed as ΔCt values. The sensitivity and reproducibility of this method was previously described^36^,^37^.

### Effects of TEX on CD69 expression on activated CD4^+^ T cells

CD4^+^ Tconv were freshly harvested from PBMC of NCs and were incubated with anti-CD3/anti-CD28 Ab-coated beads and IL-2 (150 U/ml) for 4 h. They were then co-incubated with TEX (50 ug protein) or medium in an atmosphere of 5% CO_2_ in air at 37 °C for 16 h. The cells were harvested and stained for flow cytometry using labeled Abs specific for CD4 and CD69 as described above. Flow cytometry was performed with gates set on CD4^+^ Tconv to determine their frequency and the CD69 expression level (MFI) on the T cell surface.

### Effects of TEX on CD39 protein expression levels and adenosine production in Treg

CD4^+^ CD39^+^ Treg were isolated from PBMC obtained from NCs, placed in wells of 96-well plates at the concentration of 10^6 ^cells /well and co-incubated with TEX (10 ng protein/well) in the presence of exogenous ATP (20 nM) for various time periods. Control wells contained TEX or Treg alone. Supernatants were collected and processed for mass spectrometry as previously described^12^. Cells were harvested and stained for expression of CD39 and CD73 proteins by flow cytometry as described above.

### Statistical analysis

The mRNA expression data were not normalized to *GAPDH*. Expression of this gene was variably altered by TEX, and *GADPH* was treated as any other gene. Paired analyses compared changes in mRNA expression levels in T cells co-incubated with or without exosomes. To display the data and illustrate mRNA expression levels or changes in mRNA expression levels, unsupervised heat maps were constructed. Clusters were identified by agglomerative hierarchical clustering with a complete linkage. Analysis of variance was conducted to test for effects of the following factors on mRNA expression levels: (a) the T cell phenotype (CD8^+^, CD4^+^, CD4^+^ CD39^+^); (b) the T cell activation status (activated, resting); and (c) the exosome source (tumor = TEX and DC = DEX). Initially, the interaction between activation and the source was evaluated and found to be not significant at p >0.05. Thereafter, only additive effects were tested. Fold differences in gene expression levels were calculated according to the following formula: 2^**^**^(Ct(PBS) – Ct(TEX)). The mean ΔCt values for gene expression in all tested T cell subsets were calculated using the formula: ΔCt = (Ct value for PBS - Ct value for TEX). Fluorescence intensity was calculated as MESF units and the data are presented as mean values ± SD. The p values <0.05 are considered significant.

## Additional Information

**How to cite this article**: Muller, L. *et al*. Tumor-derived exosomes regulate expression of immune function-related genes in human T cell subsets. *Sci. Rep*. **6**, 20254; doi: 10.1038/srep20254 (2016).

## Supplementary Material

Supplementary Information

## Figures and Tables

**Table 1:** Analysis of the factors (T cell type, T cell activation and exosome source (TEX vs. DEX) that could influence variation in gene expression upon co-incubation with exosomes^a^.

Gene	T cell	Activation	Exosome Source
*GAPDH*			
*CD25*		***	
*IL-2*	***		
*CD26*	***		
*TGFb*		*	
*IL-10*		**	
*PD-1*	**		
*PDL-1*			*
*CD40L*	***		
*Fas*			
*CD39*	***	**	
*FOXP3*	***	***	*
*CD73*	***	***	
*A1R*		**	
*A2AR*		**	
*A2BR*		**	
*A3RV1&V2*	**	**	
*A3RV1&V3*		**	
*COX2*			
*CTLA4*	***	***	
*PTGES*	***	***	
*ZAP70*		**	
*ADCY7*		***	
*FasL*		***	

^a^The three-way ANOVA analysis of mean gene expression for the 24 genes measured in resting and activated T cell subsets (CD4^+^ Tconv, CD8^+^ T cells and CD4^+^ CD39^+^ Treg) obtained from three different donors. All T cells were co-incubated with TEX, DEX or PBS. In comparing effects of TEX vs. DEX, mean Ct values for each factor are evaluated over all other factors.

*p < 0.05; **p < 0.01; ***p < 0.001.
