# Supplementary material for: Tumor-derived exosomes regulate expression of immune function-related genes in human T cell subsets
Source: Sci Rep. 2016 Feb 4;6:20254. doi: 10.1038/srep20254 (PMC4740743; doi:10.1038/srep20254)
Supplement: Supplementary Information [file srep20254-s1.pdf]

Supplementary Information

Tumor-derived exosomes regulate expression of immune function-related genes in human T cell subsets

Laurent Muller, Masato Mitsuhashi, Patricia Simms, William E. Gooding, and Theresa L. Whiteside

Supplementary Table 1: Primer sequences used in this study

**Gene**	**Forward (5'-3')**	**Reverse (5'3')**
ADCY7	TGGAGAACTTGGGAAAATCCA	GCCACGGCATTCACAAGAGT
A1R	GCCTTTGCACATCCTCAACTG	AGGTAAGGATGCTGGGCTTGT
A2AR	TCGGTTGTGAATCCCTTCATC	CGAATGATCTTGCGGAAGGT
A2BR	TCTGTCACATGCCAATTCAGTTG	TTGTGAAAAGTGTAGCGGAAGTCT
A3R V1&V2	CATGCCTTTGGCCATTGTT	GCAAGTCATAAAAAGGCAGCTGTA
A3R V1&V3	GCAAGGCTCCCAAAGTTGTC	ATCAGTATGCAAATGATGAGAATGG
CD25	CAGAAGTCATGAAGCCCAAGTG	GGCAAGCACAACGGATGTCT
CD26	GGAACAGCAGATGATAACGTTCAC	GGAAATCCACTCCAACATCGA
CD39	CCCAGCTGAGCAACCATTGT	GACCAGGGAGAATAGAACCATGA
CD40L	CCACAGTTCCGCCAAACCT	CACCTGGTTGCAATTCAAATACTC
CD73	GCCCAGTTATGACCCTCTCAAA	CTGGAACCCATCTCCACCAT
COX-2	GTCTCTCATCTGCAATAACGTGAAG	CCGGAGCGGGAAGAACTT
CTLA4	CACTGAGGTCCGGGTGACA	GTAGGTTGCCGCACAGACTTC
Fas	TGGCATCAACTTCATGGAAAGA	GCAAGAGTACAAAGATTGGCTTTTT
FasL	TGGCAGCATCTTCACTTCTAAATG	GAAATGAGTCCCCAAAACATCTCT
FOXP3	CACCTACGCCACGCTCATC	AAGGCAAACATGCGTGTGAA
GAPDH	CCCACTCCTCCACCTTTGAC	CATACCAGGAAATGAGCTTGACAA
IL-2	GAACTAAAGGGATCTGAAACAACATTC	TGTTGAGATGATGCTTTGACAAAA
IL-8	TGCTAAAGAACTTAGATGTCAGTGCAT	TGGTCCACTCTCAATCACTCTCA
IL-10	GCCATGAGTGAGTTTGACATCTTC	GATTTTGGAGACCTCTAATTTATGTCCTA
JAK3	CCTGTACGAGCTCTTCACCTACTG	ATCCCATCATCCGCAGGAA
PD-1	CTCAGCCGTGCCTGTGTTC	GGAAAGACAATGGTGGCATACTC
PDL1	TCCAAGAGAGAGGAGAAGCTTTTC	GCTGTATGGTTTTCCTCAGGATCT
PTGES	CACGCTGCTGGTCATCAAGAT	TCTTCTTCCGCAGCCTCACT
STAT3	GCCAGAGAGCCAGGAGCAT	GGTGTCACACAGATAAACTTGGTCTT
TGFβ1	CTGCTGAGGCTCAAGTTAAAAGTG	TGAGGTATCGCCAGGAATTGT
ZAP70	CATGAGTGACTGCTGGATCTACAA	GCTGGCCAGGCTGTAGTAACA

Supplementary Figure 1: Changes in mRNA expression levels of the *IL-8* gene in activated CD4+Tconv, CD8+ T cells and Treg after co-incubation with TEX are dependent on TEX dose, T-cell type and the TEX

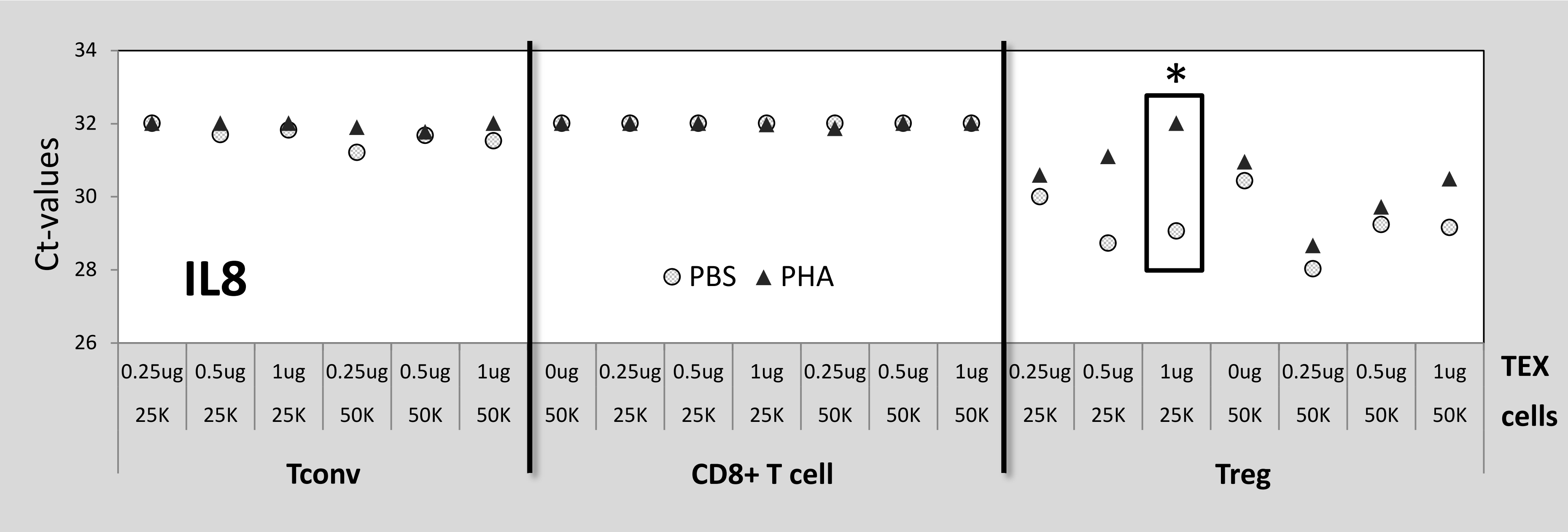

Supplementary Figure 1. Changes in mRNA expression levels of the *IL-8* gene in activated CD4+Tconv, CD8+ T cells and Treg after co-incubation with TEX are dependent on TEX dose, T-cell type and the TEX : T cell ratio. Isolated T cell subsets were activated with PHA and different numbers of T cells were co-incubated with increasing doses of TEX or PBS for 24h at 37^0 C. mRNA expression levels of the *IL-8* gene were measured as described in Methods. TEX decreased expression of mRNA (higher Ct values at p=0.015) only in Treg at the concentration of 1ug/ml per 25,000 cells.
